# Towards the first 90: impact of the national HIV self‐test program on case finding and factors associated with linkage to confirmatory diagnosis in Taiwan

**DOI:** 10.1002/jia2.25897

**Published:** 2022-03-24

**Authors:** Yen‐Fang Huang, Yu‐Ching Huang, Yi‐Chun Lo, Carl Latkin, Hsun‐Yin Huang, Chia‐Chi Lee, Li‐Chern Pan, Hsu‐Sung Kuo

**Affiliations:** ^1^ Centers for Disease Control Ministry of Health and Welfare Taipei Taiwan; ^2^ Research Center for Epidemic Prevention and One Health National Yang Ming Chiao Tung University Taipei Taiwan; ^3^ Department of Health Behavior and Society Bloomberg School of Public Health Johns Hopkins University Baltimore Maryland USA; ^4^ Graduate Institute of Biomedical Optomechatronics Taipei Medical University Taipei Taiwan

**Keywords:** HIV epidemiology, HIV self‐test, HIV testing, intervention, men who have sex with men, public health

## Abstract

**Introduction:**

Being aware of one's HIV‐positive status can help reduce unprotected sex and promote early treatment seeking. Therefore, HIV self‐test (HIVST) programs may help control the HIV epidemic by case finding. The aims of this study were to determine the effect of HIVST programs on HIV case finding, time to confirmatory diagnosis and factors associated with linkage to confirmatory diagnosis in Taiwan.

**Methods:**

The Centers for Disease Control in Taiwan initiated HIVST programs and imported 78,000 self‐test kits in 2017 and 2019. Clients paid 7 US dollars for a self‐test kit at facilities, vending machines or online. The programs set up an HIVST logistics management system; each kit had a unique barcode for monitoring the programs because purchases were anonymous. When clients provided their test results with photo barcodes online or at HIV/AIDS‐designated hospitals, they received full monetary reimbursement. We conducted a quasi‐experimental interrupted time‐series (ITS) analysis that covered a period of 60 months from 2015 to 2019. We enrolled a retrospective cohort of reported HIV cases with initial positive results from HIVST programs between March 2017 and July 2020.

**Results:**

The ITS analysis included data from 10,976 reported HIV cases from 2015 to 2019. The HIVST‐positive cohort included 386 reported HIV cases, of whom 99.7% were males and 97% were men who have sex with men (MSM); the median age was 28 years. The ITS analysis showed a positive slope change in the number of reported HIV cases immediately in the beginning implementation month (coefficient: 51.09 in 2017 and 3.62 in 2019), but there was a significant decrease over time. It was a negative slope change by 9.52 cases per month in 2017 and 5.56 cases per month in 2019. In the HIVST‐positive cohort, three of five individuals linked to HIV confirmatory diagnosis within 1 month after a positive self‐test result, and an early linkage to confirmatory diagnosis was associated with HIVST disclosure (adjusted OR = 6.5; 95% CI: 3.9–10.6).

**Conclusions:**

HIVST programs were associated with an increase in HIV case finding. Our findings suggest that countries with a high incidence of HIV among MSM populations should offer multichannel HIVST services.

## 1 INTRODUCTION

The World Health Organization has endorsed HIV self‐testing (HIVST) to reach the 95‐95‐95 treatment target and end AIDS by 2030 [1]. Several reports of successful implementation of free HIVST programs to improve the rate of HIV testing through outreach programs and door‐to‐door distribution in high‐prevalence countries have been published [2, 3, 4, 5, 6]. Recently, in Asia, some countries, including Thailand and Vietnam, have started HIVST pilot programs or studies [7, 8, 9], but most countries remain in the pre‐implementation stage [10, 11, 12, 13, 14]. In Western countries, men who have sex with men (MSM) tend to utilize HIVST through free online ordering or outreach services [15, 16, 17, 18]. However, these studies rarely measured the association between HIVST access and the trends of annual new HIV diagnosis at the population level. In addition, review articles have indicated that the measurement of linkage to confirmatory diagnosis for people who received initial positive results on an HIVST in past studies has been limited by using only self‐reported data [1, 19, 20].

In Taiwan, MSM have accounted for the majority (70–85%) of annual new HIV diagnoses since 2008 [21, 22]. The estimated HIV prevalence was 27,455 among the MSM population in 2019 [23]. One Taiwanese study also showed that MSM were the major contributing group (odds ratio [OR] 11.54) for HIV positive at anonymous testing sites [24]. Taiwan's government has established HIV/AIDS‐designated clinics/hospitals and provided free antiretroviral therapy (ART) for people with HIV since 1997 [22]. The last two 90 treatment targets reached 84 and 88 in 2016 [25]. However, an estimated one in four persons living with HIV were unaware of their infection status in 2016 [25]. In response, national HIVST programs were launched in 2017 and 2019 to enhance the accessibility of HIV testing in Taiwan, particularly for the MSM populations. These programs provided an opportunity to evaluate the effect of HIVST on HIV case finding during an extensive HIV epidemic that has greatly affected MSM in this country. The aims of this study were to explore the following: (1) the impact of HIVST programs on the HIV case finding, (2) the time from initial positive HIVSTs to confirmatory diagnosis and (3) the factors related to time to confirmatory diagnosis among those with initial positive HIVSTs.

## 2 METHODS

### 2.1 Study design

This research included two studies. In the population‐level study, we conducted a quasi‐experimental interrupted time‐series (ITS) analysis that covered a period of 60 months, including two periods prior to the two programs (January 2015–February 2017 and January 2018–November 2018) and two periods of 23 months during implementation programs (March 2017–December 2017 and December 2018–December 2019).

We also enrolled a retrospective cohort of persons living with HIV (PLHIV) with initial positive results on HIVSTs between March 2017 and July 2020 in this individual‐level study. All data were fully anonymized prior to the analysis. The Institutional Review Board Committee of the Taiwan Centers for Disease Control (TCDC) approved the research (No: IRB‐106302 and IRB‐108301) and waived the informed consent.

### 2.2 Intervention

The TCDC imported 18,000 HIV oral self‐testing kits for the first program in 2017 and 60,000 kits for the second program in 2019 through special permission from the Taiwan Food and Drug Administration since there is no government‐approved HIV self‐testing kit available in Taiwan. Clients could obtain the self‐test kit through three avenues: at a facility, from a vending machine or online with a pay‐at‐pickup option. The program limited the maximum amount available for online purchasing to 7000 in 2017 due to contract of shipment limit, but did not set a maximum amount in 2019. The duration of the two programs was from March to December 2017 and December 2018 to December 2019.

The program also produced videos on its website to demonstrate how to use the kit and established a 24‐hour hotline for counselling about the HIVST. A flyer containing the list of designated clinics/hospitals list for confirmatory HIV diagnosis was included in the kit package. Clients paid 7 US dollars (200 NT dollars) to obtain the kit. Test results could be obtained in 20 minutes. When clients logged their test results and the kit's photo barcode on the website or went to an HIV/AIDS‐designated clinic/hospital and showed the kit's barcode, they received full monetary reimbursement. If the result was logged as positive, the webpage would automatically pop up an offer for assistance. When clients chose to log their contact information, it would send the notification to case managers so that they could contact clients to schedule an appointment. The 2017 program established an online consumer survey to invite the clients who logged their test results to fill out survey.

Most facility‐based sites were local health stations around the country and gay health centres in major cities, while others were pharmacies and gay‐friendly stores. The vending machines were located at sites popular with the MSM population, including saunas and gay health centres. Chain convenience stores have provided pay‐at‐pickup services for the past 15 years in Taiwan. Clients can access the TCDC website (https://hiva.cdc.gov.tw/oraltest) to log a nickname, mobile phone number and designate which store to pick up the kit. After the kit package was delivered to the designated store, a text message notified the client. At the counter of convenience stores, clients provided the clerk the last three digits of their cellphone number and paid the equivalent of 8 US dollars plus the delivery fee to obtain a kit.

### 2.3 Participants and sources of data

The ITS analysis included data from the HIV/AIDS reporting/case management system. The HIVST‐positive cohort was recruited according to the kit barcodes that clients provided or the results in two databases (HIV reporting system and HIV logistics management system) linked by phone number. The TCDC set up an HIVST logistics management system; each kit had a unique barcode for monitoring the HIVST programs because of anonymous purchasing.

We set several channels to identify cases with positive results on the HIVST. One was online anonymous self‐reporting of the logged barcode with the test results in the HIVST logistics management system. However, we did not have further information about their dates of HIV diagnosis from online self‐reporting. Therefore, the TCDC cooperated with HIV/AIDS‐designated hospitals/clinics and local health bureaus to ask confirmed patients about their HIVST experience and barcode number in routine case interviews. Then, we matched the barcodes between online self‐reporting and routine case interviews and obtained the dates of receiving the kits and HIV diagnosis. For the online purchase group, the clients provided their phone numbers; therefore, the third source linked the results in the HIVST logistics management system with those in the HIV/AIDS management system by cellphone number to identify confirmed cases that had used HIVST but not disclosure in the two above ways. The demographic data of the HIVST‐positive cohort were from the HIV/AIDS reporting/case management system.

### 2.4 Outcomes and measures

The primary outcomes were the immediate step change and slope change of the number of reported HIV cases per month between the non‐implementation period and implementation period. Our study refers to observations before the HIVST program as the “non‐implementation period” and those after the program as the “implementation period.”

The secondary outcomes included the time to confirmatory diagnosis and factors associated with early confirmation diagnosis in the HIVST‐positive cohort. The time interval to diagnosis was calculated as the date of obtaining HIVST kits (the purchases date in the HIVST logistic system) to the date of HIV reported. If someone purchased multiple kits, we selected the most recent date of HIVST kit purchase. We defined an early diagnosis as a confirmed HIV diagnosis within 1 month of the date of obtaining HIVST kits as an initial positive result on HIVST. We also defined the HIVST non‐disclosure group as cases from the databases that were linked by phone number.

### 2.5 Statistical analysis

The number of reported cases from HIVST programs will be underestimated due to anonymity. Therefore, we used an ITS analysis to evaluate the impact of the HIVST programs on case finding at the population level. ITS analysis examines temporally ordered data to determine whether interventions produced a reliable change in data while also allowing models to account for baseline levels and trends present in the data [26, 27, 28, 29].

To assess the factors associated with early linkage to confirmatory diagnosis among those with initial positive results from the HIVST, we constructed multiple logistic regression models. The adjusted models controlled for subject age, sex, education, program year and transmission category. We tested for collinearity with a correlation matrix but did not detect any collinearity issues. In addition, we also used Hosmer and Lemeshow's goodness‐of‐fit test to examine the fit of the model. All analyses were conducted using SAS version 9.4.

## 3 RESULTS

### 3.1 Description of the HIVST population

**Figure 1:**
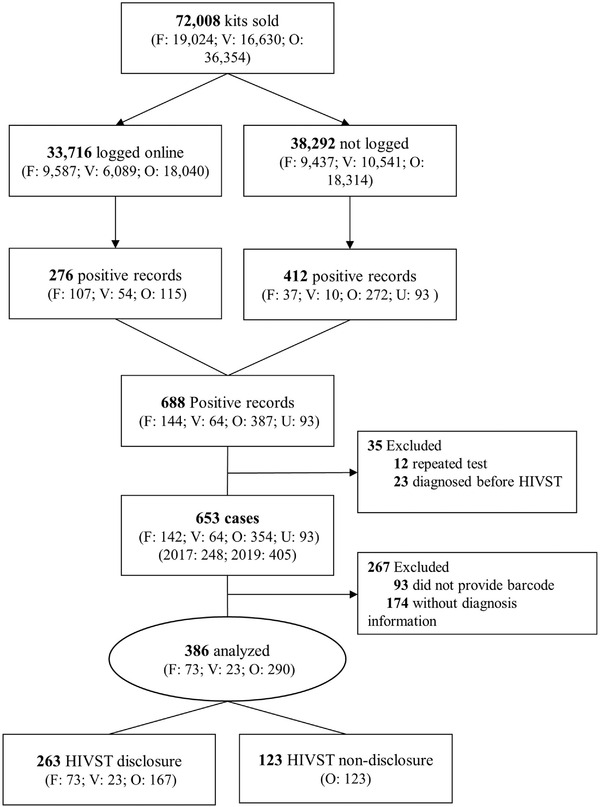
Study sample recruitment process. Abbreviations: F, facilities; O, online; U, HIVST disclosure to health workers but without barcodes (unknown purchasing channels); V, vending machines.

Overall, approximately half (33,716; 47%) of the 72,008 kits had logged barcodes and reported test results. The number and response rate of logged barcodes and testing results increased yearly from 6935 (39%) in 2017 to 26,781 (49%) in 2019. The number and the total rate of initial positive results among individuals who reported their test results (online self‐reported) was 276/33,716 (0.8%); this value decreased by program year from 1.3% in the 2017 program to 0.7% in the 2019 program. The initial positivity rates associated with different HIVST channels, including health facilities, vending machines and online channels, were 1.8%, 1.0% and 1.1% in 2017, and 0.9%, 0.9% and 0.6% in 2019, respectively (Table 1 and Figure 1).

**Table 1:** Description of the HIVST programs

Variables		Kits sold	Sites	Average sales per site	Logged online	Logged response rate (%)	Self‐reported initial positive results	Initial positivity rate (%)
Total	All	72,008			33,716	46.8	276	0.8
	Facilities	19,024	432	44	9587	50.4	107	1.1
	Vending machines	16,630	42	396	6089	36.6	54	0.9
	Online	36,354			18,040	49.6	115	0.6
2017	All	17,788			6935	39	89	1.3
	Facilities	5854	215	27	2265	38.7	41	1.8
	Vending machines	5071	23	220	1549	30.5	15	1
	Online	6863			3121	45.5	33	1.1
2019	All	54,220			26,781	49.4	187	0.7
	Facilities	13,170	359	37	7322	55.6	66	0.9
	Vending machines	11,559	29	399	4540	39.3	39	0.9
	Online	29,491			14,919	50.6	82	0.6

The number and response rate in the customer survey were 3730 (21%) in 2017. Of the participants, 90% were men more than 12 years of education. The sexual orientation of the participants included 2136 (57%) homosexual, 469 (13%) bisexual and 1125 (30%) heterosexual. The median age at the date of diagnosis was 27 years (range: 24–32 years). More than half (53%) of respondents obtained the kits online (Appendix S1).

We also identified 412 initial HIV‐positive results through case interviews or phone number linkages among the sub‐population that did not log their results online. A total of 688 initial HIV‐positive results were identified among the HIVST programs. Deleting 12 repeated barcodes and 23 barcodes for which the test date was after the reporting date, the number of initial positive cases was 653 (Figure 1). The rate of initial positive results among the 72,008 individuals was 0.9%, 1.4% in 2017 and 0.8% in 2019, respectively.

According to the enrolment criteria of having the two time points as the date of purchase to the date of reporting for calculating the time to linkage to confirmatory diagnosis, we excluded 174 barcodes without associated confirmatory HIV results and 93 cases with positive HIV confirmatory results without associated barcodes. A total of 386 individuals were included in the analysis of time to linkage to confirmatory diagnosis (Figure 1).

### 3.2 Description of the HIVST‐positive cohort

The HIVST‐positive cohort of 386 individuals included 385 (99.7%) males and 1 (0.3%) female. Most of the subjects (97%) were MSM. The median age at the date of diagnosis was 28 years (range: 24–32 years). In total, 78% of participants had an education level higher than 12 years at the date of diagnosis. In nearly one‐third (118, 30%) of participants, the HIVST was the first HIV test received (Table 2).

**Table 2:** Baseline characteristics of the HIVST‐positive cohort

Characteristic	Total	Time to confirmatory diagnosis		*p*‐Value
		<30 days	> = 30 days	
	*N* = 386 (%)	*n* = 230 (%)	*n* = 156 (%)	
Program year				
2017	138 (35.8)	70 (50.7)	68 (49.3)	0.008
2019	248 (64.2)	160 (64.5)	88 (35.5)	
Median age (Q1, Q3)	28 (24, 32)	28 (24, 32)	27 (24, 32)	0.393
Sex				
Female	1 (0.3)	1 (100)	–	–
Male	385 (99.7)	229 (59.5)	156 (40.5)	
Transmission category				
MSM	376 (97.4)	225 (59.8)	151 (40.2)	0.531
Heterosexual	10 (2.6)	5 (50)	5 (50)	
Education				
≦12 years	84 (21.8)	54 (64.3)	30 (35.7)	0.321
>12 years	302 (78.2)	176 (58.3)	126 (41.7)	
Ever tested for HIV				
No	118 (30.4)	69 (58.5)	49 (41.5)	0.768
Yes	268 (69.6)	161 (60.1)	107 (39.9)	
HIVST disclosure				
No	123 (31.7)	37 (30.1)	86 (69.9)	<0.001
Yes	263 (68.3)	193 (73.4)	70 (26.6)	

By 31 July 2020, a date which allowed at least a 1‐year follow‐up after HIV diagnosis, most patients (98%) were on ART, with half of the patients receiving ART within 1 month of diagnosis. A total of 355 (92%) individuals progressed to viral load suppression after treatment.

### 3.3 The impact of the HIVST program on the annual new HIV reported

**Figure 2:**
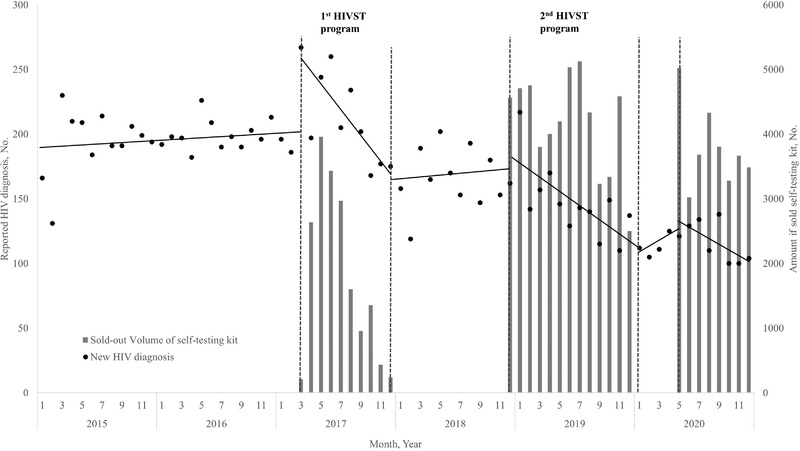
The impact of the HIVST programs on the case finding curve according to ITS analysis.

From 2015 to 2020, a total of 12,362 HIV cases were reported. The distribution of annual reported cases by year was 2325 in 2015, 2394 in 2016, 2510 in 2017, 1990 in 2018, 1751 in 2019 and 1392 in 2020. The ITS analysis results showed that there was a small increase in the average monthly number of diagnoses before program implementation. However, we observed a significant change in the trend of the reported cases from the non‐implementation period to the implementation period. The 2017 program had a significant positive step change at the beginning moment from the non‐implementation period (before March 2017) to the implementation period (b = 51.09; 95% CI = 18.78–83.39; *p* = 0.003); in contrast, there was a significant decreasing slope change over time during the implementation period (b = –9.52; 95% CI = –13.89 to –5.14; *p* = <0.0001). In the subsequent non‐implementation period (between January and November 2018), the number of reported cases showed an increasing but not significant trend. For the second program, a positive step change occurred as well, but the change at the beginning moment from the non‐implementation period was not significant (between January and November 2018) to the implementation period (b = 3.62; 95% CI = –21.48 to 28.73; *p* = 0.766); moreover, there was a significantly decreased slope change (b = –5.6; 95% CI = –10.32 to –0.79; *p* = 0.024) (Table 3 and Figure 2).

**Table 3:** The impact of the HIVST programs on the new HIV diagnosis according to ITS analysis

		Constant	No policy implementation	Immediate effect of policy implementation	Change in trend after policy implementation
First HIVST program (Mar–Dec 2017)	Coefficient	190.32	0.47	51.09	–9.52
	95% CI	166.8–213.89	–0.92 to 1.86	18.784–83.39	–13.89 to –5.14
	*p* value	<0.001	0.69	0.003	<0.001
Second HIVST program (Dec 2018–Dec 2019)	Coefficient	161.73	0.91	3.62	–5.56
	95% CI	133.33–190.12	–2.29 to 4.76	–21.48 to 28.73	–10.32 to –0.79
	*p* value	<0.001	0.628	0.766	0.024

### 3.4 Time to HIV confirmatory diagnosis

We found that the peak time of HIV confirmatory test was during the first week after purchasing the self‐test kit; 92 (27%) participants who obtained an initial positive result had their diagnosis confirmed within the first week after purchasing the self‐test kit. Among the other participants, the durations to confirmatory diagnosis varied from 2 to 123 weeks.

**Figure 3:**
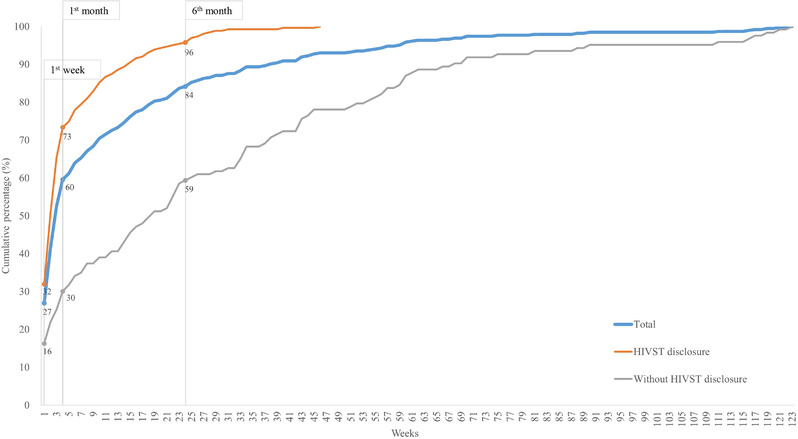
Time to HIV confirmatory diagnosis.

In the cumulative calculation, 60% of participants with an initial positive result who linked HIV confirmatory diagnosis during the first month after purchasing the self‐test kit. Overall, 263 cases disclosed HIVST purchases, among whom 193 (73%) linked HIV confirmatory diagnosis within 1 month after purchasing the self‐test kit in the HIVST disclosure group. In the non‐disclosure group, the percentage of early linkage confirmatory diagnosis was 30% (Figure 3).

### 3.5 Early to confirmatory diagnosis associated with HIVST disclosure

Table 4 shows the results of the unadjusted and adjusted logistic regression models in which the early linkage confirmatory diagnosis group served as the reference. The likelihood of early diagnosis among those who disclosed their HIVST experience was significantly higher than the likelihood of those who did not disclose their HIVST experience, as indicated in both the adjusted (adjusted OR = 6.45; 95% CI = 3.92–10.58; *p* <0.0001) and unadjusted models (OR = 6.41; 95% CI = 4.00–10.28; *p* <0.0001). Age, education and transmission route were not significantly associated with time to diagnosis.

**Table 4:** Logistic regression models of early linkage to confirmatory diagnosis^a^ (*N* = 386)

Characteristic	Odds ratio (95% CI)	*p*‐Value	Adjusted odds ratio (95% CI)	*p*‐Value
Program year				
2017	1 [Reference]		1 [Reference]	
2019	1.77 (1.16–2.70)	0.008	1.26 (0.79–2.03)	0.333
Age	1.00 (0.97–1.03)	0.812	1.00 (0.97–1.04)	0.858
Transmission route				
Heterosexual	1 [Reference]		1 [Reference]	
MSM	1.49 (0.42–5.24)	0.534	1.19 (0.29–4.96)	0.812
Education				
> = 12 years	1 [Reference]		1 [Reference]	
>12 years	1.29 (0.78–2.13)	0.322	1.29 (0.74–2.24)	0.370
Ever HIV testing				
Yes	1 [Reference]		1 [Reference]	
No	0.94 (0.60–1.45)	0.767	1.32 (0.80–2.19)	0.280
HIVST disclosure				
No	1 [Reference]		1 [Reference]	
Yes	6.41 (4.00–10.28)	<0.001	6.45 (3.92–10.58)	<0.001

^a^ The reference category is those whose time from HIVST to linkage confirmatory diagnosis was less than 30 days.

## 4 DISCUSSION

The ITS analysis of the average number of monthly diagnoses showed an initial increase, but a subsequent decline during the implementation of the HIVST programs, which could imply the HIVST program accelerated case finding. Although our positive cohort was small, there was an increase of annual reported HIV cases in 2017 when compared to that in 2016, which was similar to the size of our positive cohort in 2017. In Taiwan, pregnant women, military draftees, prisoners, people who use drugs and individuals with sexually transmitted diseases (STDs), among others, receive routine HIV screening; the number of individuals tested is around 650,000 per year. The TCDC also cooperated with designated HIV/AIDS hospitals/clinics since 2000 and gay health centres since 2010 to provide anonymous testing and outreach services for the MSM population. The number of individuals tested per year between 2016 and 2019 plateaued at 40,000 at anonymous sites and 10,000 at gay health centres. The HIV‐positive rate in anonymous sites and gay health centres was 0.9% and 0.5% in 2017, and 0.5% and 0.6% in 2019, respectively. The HIVST program was the only new strategy implemented for HIV testing in Taiwan in 2017 and 2019, besides other HIV testing services, including outreach testing by community organizations and anonymous testing in clinical settings, were ongoing. The HIV‐positive rate in the HIVST population was similar to that at the anonymous sites and gay health centres in Taiwan. Taiwan's first 90 treatment target was also improved from 79 in 2016 to 88 in 2019 [23]. Some high‐income countries and cities, including New York City in the United States and some countries in the European Union, have established multichannel HIVST services for MSM population and achieved the first 90% target before 2020 [1, 27, 28, 29].

Overall, the trend of the reported cases in Taiwan has decreased since 2017. HIVST programs alone will not lead to decrease in this epidemic; comprehensive prevention programs and treatment strategies are also important and necessary. Early ART for viral suppression has been shown to decrease HIV transmission, impacting the trajectory of the epidemic [30, 31, 32, 33, 34]. A high proportion of PLHIV received ART in 2019 (92%) and viral suppression (95%) in Taiwan [23]. It is very likely that reduced HIV transmission among those receiving ART and viral suppression also played a significant role in controlling the epidemic after the increase in the number of cases identified. The TCDC also initiated a pre‐exposure prophylaxis (PrEP) pilot program for sexual partners of PLHIV and individuals under 30 with high‐risk behaviours (substance abuse, STDs, MSM, etc.) in 2017 and 2019. The PrEP flyer was included in the HIVST kit package, so that those who tested HIV negative could learn about this service. The number of actual participants versus the PrEP quota was 324/1000 in 2017 and 841/1500 in 2019. To sum up, comprehensive prevention programs, multichannel HIV testing services and treatment strategies may continue to decrease the HIV epidemic in Taiwan.

Our HIVST programs effectively reached the MSM population. The initial HIV positivity rate among the participants of the HIVST programs was similar to those in past studies in Western countries [15, 35, 36, 37]. Previous studies showed that significant barriers to HIV testing included stigma, confidentiality issues and inconvenience accessing a testing facility [38, 39]. In Western countries, specific populations, such as MSM, tend to accept HIV self‐testing and have high testing rates through free online ordering or outreach services [15, 16, 17]. In Taiwan, the official estimate of the MSM population is approximately 1.7% of the adult male population [40]. However, this number may be underestimated because MSM in Taiwan tend to hide their sexual identities due to social stigma and homophobia [41]. The Taiwanese government has made many efforts for gender equity, such as legal same‐sex marriage since 2019 [42]. The TCDC has long cooperated with MSM community organizations for HIV prevention. In the planning stage for the HIVST programs, these sites were chosen after discussion with stakeholders in the community.

This study is also the first report to describe the detailed time distribution from an initial positive result to confirmatory diagnosis in the HIVST population. Our results showed that most of the initial positive cases sought medical care to obtain an HIV diagnosis within 1 month after obtaining initial positive results. In Taiwan, the National Health Insurance System covers all medical fees, and people only needed to pay 5 US dollars per visit. In addition, the Taiwanese government and the National Health Insurance System have offered free access to ART for HIV‐positive individuals since 1997 [43]. Taiwan also designated HIV case managers in HIV/AIDS‐designated hospitals to provide patient‐centred care since 2004. These strategies may address barriers to obtaining a confirmatory HIV diagnosis among individuals with an initial positive result.

Previous studies have indicated that individuals were increasingly concerned about STI‐related stigma, and this concern was associated with a significantly decrease in STI‐related testing and seeking of care [38, 44, 45]. Our study indicated that cases with non‐disclosure of HIVST kit purchasing had delayed confirmatory diagnoses compared to those who disclosed kit purchasing. We hypothesized that the longer latency to confirmatory diagnosis in the HIVST non‐disclosure group may be related to HIV/STI‐related stigma issues from the HIV case managers’ observations. Other possible reasons included recall bias. However, it is important to note that 84% of cases were linked to confirmatory diagnosis within 6 months of receiving the HIVST initial positive results. In other words, HIVST programs could attract the MSM population, who may be concerned about HIV/STI‐related stigma.

Our analysis was limited to the data available in the databases and the anonymous services. Thus, it must be noted that the total number of HIVST‐positive individuals who we defined may be underestimated. In addition, ITS analysis cannot distinguish between the impact of the HIVST intervention and other prevention programs. Despite these limitations, our study has some unique strengths. Our study used multiple interlinked data sources by applying a barcode system to evaluate the effects of HIVST programs while allowing the participants of the HIVST programs to remain anonymous. We collected HIV‐positive participants’ personal demographic and related diagnosis information. Most other published studies on HIVST programs did not obtain such detailed information.

## 5 CONCLUSIONS

We found that nationwide multichannel HIVST strategies improved the HIV case finding. Working with the community in hidden populations to identify key methods for accessing testing and sufficient coverage is also very important. HIVST may also be useful to help identify an outbreak and more quickly control it. The TCDC has routinely implemented the HIVST program as one of the national strategies. Currently, there is no limit on the number of online purchases. In addition, a membership service was also implemented to remind of individuals to receive regular HIV testing. During the COVID‐19 pandemic, HIVSTs provide individuals with continued testing services. Our findings, on the whole, suggest that countries with a high incidence of HIV among MSM populations should offer multichannel HIVST services.

## COMPETING INTERESTS

The authors have no competing interests.

## AUTHORS’ CONTRIBUTIONS

YFH and YCH designed the study. YFH wrote the first draft. YCH performed the statistical analyses. HYH and CCL contributed to data collection. YFH, YCL, CL, LCP and HSK interpreted the results and wrote the manuscript, and all authors approved the final draft.

## DISCLAIMER

The Taiwan Centers for Disease Control had no role in the design, analysis or writing of this manuscript or the decision to submit the paper for publication. The contents of this paper are solely the responsibility of the authors and do not necessarily represent the official views of the Taiwan Centers for Disease Control.

## Supporting information


**Appendix S1** The number and response rate in the customer survey in 2017 (N=3,730).
