# Supplementary material for: Towards the first 90: impact of the national HIV self‐test program on case finding and factors associated with linkage to confirmatory diagnosis in Taiwan
Source: J Int AIDS Soc. 2022 Mar 24;25(3):e25897. doi: 10.1002/jia2.25897 (PMC8944217; doi:10.1002/jia2.25897)
Supplement: Supplementary file 1 — Appendix S1 The number and response rate in the customer survey in 2017 (N=3,730). [file JIA2-25-e25897-s001.docx]

| **Variable** | **N** | **%** |
| --- | --- | --- |
| **Gender** |  |  |
| Male | 3,341 | 89.6 |
| Female | 385 | 10.3 |
| Transgender | 4 | 0.1 |
| **Sexual orientation** |  |  |
| MSM | 2,136 | 57.3 |
| Heterosexual | 1,125 | 30.2 |
| Bisexual | 469 | 12.6 |
| **Age Group** |  |  |
| 15-24y | 1,028 | 27.6 |
| 25-34y | 2,067 | 55.4 |
| 35-44y | 549 | 14.7 |
| >=45y | 86 | 2.3 |
| median (Q1,Q3) | 27 (24,32) | |
| **Education** |  |  |
| <=12yrs | 384 | 10.3 |
| >12yrs | 3,346 | 89.7 |
| **HIVST channels** |  |  |
| Facilities | 982 | 26.3 |
| Vending machines | 762 | 20.4 |
| Online | 1,986 | 53.2 |
|  |  |  |

Appendix 1 The number and response rate in the customer survey in 2017 (N=3,730).
